# Hospital nurses’ knowledge of adult cardiopulmonary resuscitation: a systematic review

**DOI:** 10.1186/s12912-026-05483-1

**Published:** 2026-10-17

**Authors:** Aintzane Beti-Abad, Lorena Hernando-Gorgojo, Nuria De-Ríos-Briz, Joan Blanco-Blanco, Beverly Holland, Verónica Tíscar-González

**Affiliations:** 1 Biobizkaia Health Research Institute, Plaza de Cruces 12, Barakaldo, 48903 Spain; 2 Osakidetza Basque Health Service, Bilbao-Basurto Integrated Health Organisation, Avenida Montevideo 18, Bilbao, 48013 Spain; 3 University of Deusto, Avenida de las Universidades 24, Bilbao, 48007 Spain; 4 Academy of Nursing Sciences of Bizkaia, Alameda San Mamés 37, Bilbao, 48010 Spain; 5 Bioaraba Health Research Institute, Care Research Group, José Atxotegi, Vitoria-Gasteiz, 01009 Spain; 6 Osakidetza Basque Health Service, Vitoria-Gasteiz Nursing School, José Atxotegi, Vitoria-Gasteiz, 01009 Spain; 7 Osakidetza Basque Health Service, Barakaldo-Sestao Integrated Health Organisation, Avenida Miranda 5, Barakaldo, 48902 Spain; 8 Nursing and Physiotherapy Department – GESEC, University of Lleida, Montserrat Roig 2, Lleida, 25198 Spain; 9 Biomedical Research Institute of Lleida – Dr. Pifarré Foundation, IRB Lleida, GRECS, Alcalde Rovira Roure 80, Lleida, 25198 Spain; 10 CIBERFES – Biomedical Research Networking Centre in Frailty and Healthy Ageing, Monforte de Lemos 3–5, Madrid, 28029 Spain; 11 Providence Holy Cross Medical Center, 15031 Rinaldi Street, Mission Hills, CA 91345 USA

**Keywords:** Cardiopulmonary resuscitation, In-hospital cardiac arrest, Hospital nurses, Knowledge, Systematic review

## Abstract

**Background:**

Hospital nurses are often among the first healthcare professionals to recognize in-hospital cardiac arrest, activate the emergency response system, and initiate cardiopulmonary resuscitation. Adequate and current knowledge is therefore essential for timely, guideline-based care. However, studies assessing nurses’ knowledge of adult cardiopulmonary resuscitation vary widely in populations, settings, assessment instruments, and criteria used to define adequate knowledge. This systematic review aimed to synthesize international evidence on hospital nurses’ objectively assessed knowledge of adult cardiopulmonary resuscitation in the context of in-hospital cardiac arrest.

**Methods:**

A systematic review of observational studies was conducted and reported according to the Preferred Reporting Items for Systematic Reviews and Meta-Analyses 2020 statement. PubMed, Embase, Scopus, Web of Science, CINAHL, LILACS Plus, PsycINFO and the Cochrane Central Register of Controlled Trials (CENTRAL) were searched on March 15, 2026, for studies published from January 1, 2010 onward, with no language restrictions. Eligible studies quantitatively assessed adult cardiopulmonary resuscitation knowledge among hospital nurses. Two reviewers independently performed study selection, data extraction, and methodological quality assessment. Methodological quality was assessed using the Joanna Briggs Institute Critical Appraisal Checklist for studies reporting prevalence data. Because of substantial variability in instruments, scoring systems, and competency thresholds, findings were synthesized narratively.

**Results:**

Twenty-five studies involving 4,653 nurses from 17 countries were included. All included studies used cross-sectional designs. Knowledge assessment methods varied considerably, with instruments ranging from 7 to 32 items and covering general cardiopulmonary resuscitation, basic life support, advanced life support, or combined domains. Reporting of instrument validity and reliability was inconsistent. Competency thresholds ranged from approximately 50% to more than 85% of correct responses, while several studies used relative or study-specific classifications. Overall, hospital nurses’ knowledge was frequently reported as poor, insufficient, moderate, or average. This methodological variability precluded quantitative pooling and limited direct comparison across studies.

**Conclusions:**

Hospital nurses’ knowledge of adult cardiopulmonary resuscitation was frequently incomplete or intermediate. Interpretation was limited by variability in assessment instruments, knowledge domains, and competency thresholds. These findings support regular training, periodic assessment, and standardized, validated, role-sensitive instruments to identify knowledge gaps and guide targeted education.

**Systematic review registration:**

PROSPERO CRD420261351580.

**Clinical trial number:**

Not applicable.

**Supplementary Information:**

The online version contains supplementary material available at https://doi.org/10.1186/s12912-026-05483-1.

## Background

Cardiac arrest is the abrupt cessation of cardiac mechanical activity, confirmed by the absence of signs of circulation, and requires immediate recognition and initiation of appropriate resuscitation interventions [1]. In-hospital cardiac arrest is a major patient safety concern and remains associated with substantial mortality and neurological morbidity. Approximately 292,000 in-hospital cardiac arrests occur annually in the United States [2], while European studies have reported incidences ranging from 1.5 to 2.8 events per 1,000 hospital admissions [3].

Outcomes following in-hospital cardiac arrest are influenced by non-modifiable factors, including age, comorbidities and the severity of the underlying illness, as well as by modifiable aspects of care [4, 5]. These include early recognition, prompt initiation of high-quality cardiopulmonary resuscitation (CPR), timely defibrillation when indicated, effective activation of the hospital emergency response system and access to post-resuscitation care. Delays in initiating CPR, defibrillation and other time-sensitive interventions are associated with reduced survival [6]. A timely, coordinated and guideline-based response is therefore essential to improve clinical and neurological outcomes [7].

Nurses have a central role in the recognition and initial management of in-hospital cardiac arrest. Their continuous presence at the bedside and close monitoring of hospitalized patients mean that they are frequently among the first healthcare professionals to identify clinical deterioration, recognize cardiac arrest, activate the in-hospital emergency response and initiate CPR [8, 9]. These responsibilities extend beyond emergency departments and critical care units, as cardiac arrest may occur in any hospital setting. Consequently, nurses working in both critical and non-critical care areas require adequate and up-to-date knowledge of adult CPR.

The knowledge required to respond to cardiac arrest encompasses several domains, including recognition of cardiac arrest, activation of emergency procedures, delivery of high-quality chest compressions, appropriate ventilation, early use of a defibrillator and adherence to current resuscitation algorithms. As resuscitation science continues to evolve, international organizations such as the American Heart Association and the European Resuscitation Council regularly update their recommendations [10, 11]. Ongoing education is therefore essential to help nurses maintain up-to-date knowledge and familiarity with current evidence-based guidance [12].

Numerous studies across countries and hospital settings have assessed nurses’ knowledge of cardiac arrest and CPR. However, their findings are difficult to compare because of differences in study populations, clinical settings, educational backgrounds, resuscitation guidelines, assessment instruments and criteria used to define adequate knowledge. This methodological heterogeneity limits the interpretation and generalizability of the available evidence and makes it difficult to identify consistent knowledge gaps and educational priorities.

A systematic synthesis of this evidence is needed to determine the reported levels and domains of adult CPR knowledge among hospital nurses and to examine how this knowledge has been assessed. Such evidence may support the development of targeted continuing education programs, standardized knowledge assessments and institutional strategies to improve preparedness for in-hospital cardiac arrest.

To our knowledge, no recent systematic review has specifically synthesized international evidence on objectively assessed adult CPR knowledge among hospital nurses, while clearly distinguishing theoretical knowledge from related constructs such as attitudes, confidence, self-efficacy, practical skills, clinical performance or educational intervention outcomes.

## Methods

### Protocol and reporting

This systematic review aimed to synthesize international evidence on hospital nurses’ objectively assessed knowledge of adult cardiopulmonary resuscitation in the context of in-hospital cardiac arrest. A systematic review of observational studies was conducted. The protocol was registered in PROSPERO (CRD420261351580), and the review was reported in accordance with the Preferred Reporting Items for Systematic Reviews and Meta-Analyses (PRISMA 2020) statement [13].

### Information sources and search strategy

A comprehensive search was conducted in PubMed, Embase, Scopus, Web of Science, CINAHL, LILACS Plus, PsycINFO, and the Cochrane Central Register of Controlled Trials (CENTRAL). The search strategy was initially developed for PubMed and subsequently adapted to each database using database-specific controlled vocabulary, including MeSH terms where applicable, and free-text terms related to cardiac arrest, cardiopulmonary resuscitation, knowledge, and nursing. The database searches were conducted on March 15, 2026, and covered studies published from January 1, 2010, to the date of the search, with no language restrictions.

The date restriction was selected because the 2010 resuscitation guidelines introduced a major change in the basic life support sequence, from airway–breathing–chest compressions (A–B–C) to chest compressions–airway–breathing (C–A–B), thereby prioritizing the early initiation of high-quality chest compressions [14]. Studies based on pre-2010 recommendations were therefore considered less comparable with contemporary resuscitation guidelines. In addition, backward citation searching was conducted by screening the reference lists of all included studies in May 2026. No forward citation searching was performed. The complete search strategies for all databases are provided in Additional file 1.

### Eligibility criteria

Original quantitative observational studies were eligible when they objectively assessed nurses’ knowledge of adult CPR in hospital settings. Eligible participants were nurses involved in the care of adult hospitalized patients. Studies including mixed professional groups were eligible only when data for hospital nurses could be extracted separately.

Objectively assessed knowledge was defined as knowledge evaluated using a structured test or questionnaire. Studies were required to report an overall or extractable quantitative measure of knowledge, such as a total score, percentage of correct responses, or proportion of participants classified according to a predefined knowledge category. When studies assessed knowledge alongside other constructs, only the objectively assessed knowledge component was considered.

Studies were excluded if they focused on attitudes, practices, confidence, self-efficacy, skills, performance, or educational interventions rather than knowledge. Studies assessing only a single isolated component, such as defibrillator use or resuscitation medications, were excluded if they did not provide a broader assessment of adult CPR knowledge.

Studies conducted outside hospital settings and those focused exclusively on pediatric, neonatal, or obstetric resuscitation were excluded. Studies using combined adult and pediatric questionnaires were eligible only when adult data could be extracted separately.

Qualitative studies, case reports, case series, conference abstracts, editorials, letters, commentaries, protocols, theses, dissertations, and reviews were excluded. Intervention studies, including pre–post designs, were excluded in accordance with the prespecified observational scope of the review. Although baseline assessments may provide information on CPR knowledge, these samples may have been selected in relation to planned educational interventions and were therefore not considered directly comparable with observational estimates of nurses’ adult CPR knowledge.

### Study selection

Records were imported into Covidence. Duplicates were identified automatically and verified manually. Two reviewers independently screened titles and abstracts against the eligibility criteria. Potentially eligible reports were assessed in full text by the same reviewers, and reasons for exclusion were recorded. Disagreements were resolved through discussion or, when necessary, consultation with a third reviewer.

### Data collection

Two reviewers independently extracted data using a standardized form developed for the review. Extracted data included the first author, publication year, country, sample size, assessment instrument, knowledge outcomes, criteria used to classify knowledge levels, and previous CPR training or certification, when reported. Discrepancies were resolved through discussion. The completed dataset was subsequently reviewed by all authors. Missing or unclear information was recorded as not reported, and study authors were not contacted for additional information.

### Methodological quality assessment

Methodological quality was assessed independently by two reviewers using the nine-item Joanna Briggs Institute (JBI) Critical Appraisal Checklist for Studies Reporting Prevalence Data (2020 version) [15]. This checklist was selected because the included studies primarily used cross-sectional survey designs to describe the distribution or proportion of nurses achieving study-defined levels of CPR knowledge. Discrepancies were resolved through discussion or, when necessary, consultation with a third reviewer. No study was excluded solely on the basis of methodological quality. Appraisal findings were considered when interpreting confidence in and the consistency and comparability of the findings.

### Data analysis and synthesis

A meta-analysis was not performed because of substantial heterogeneity in the assessment instruments, scoring systems, and cut-off points used to define adequate knowledge. Findings were synthesized narratively and summarized according to assessment methods, knowledge outcomes, and study-defined competency thresholds. Previous CPR training and certification were described when reported. Given this heterogeneity and the absence of pooled estimates, reporting bias and the certainty of the overall body of evidence were not formally assessed.

## Results

### Search results

**Fig. 1:**
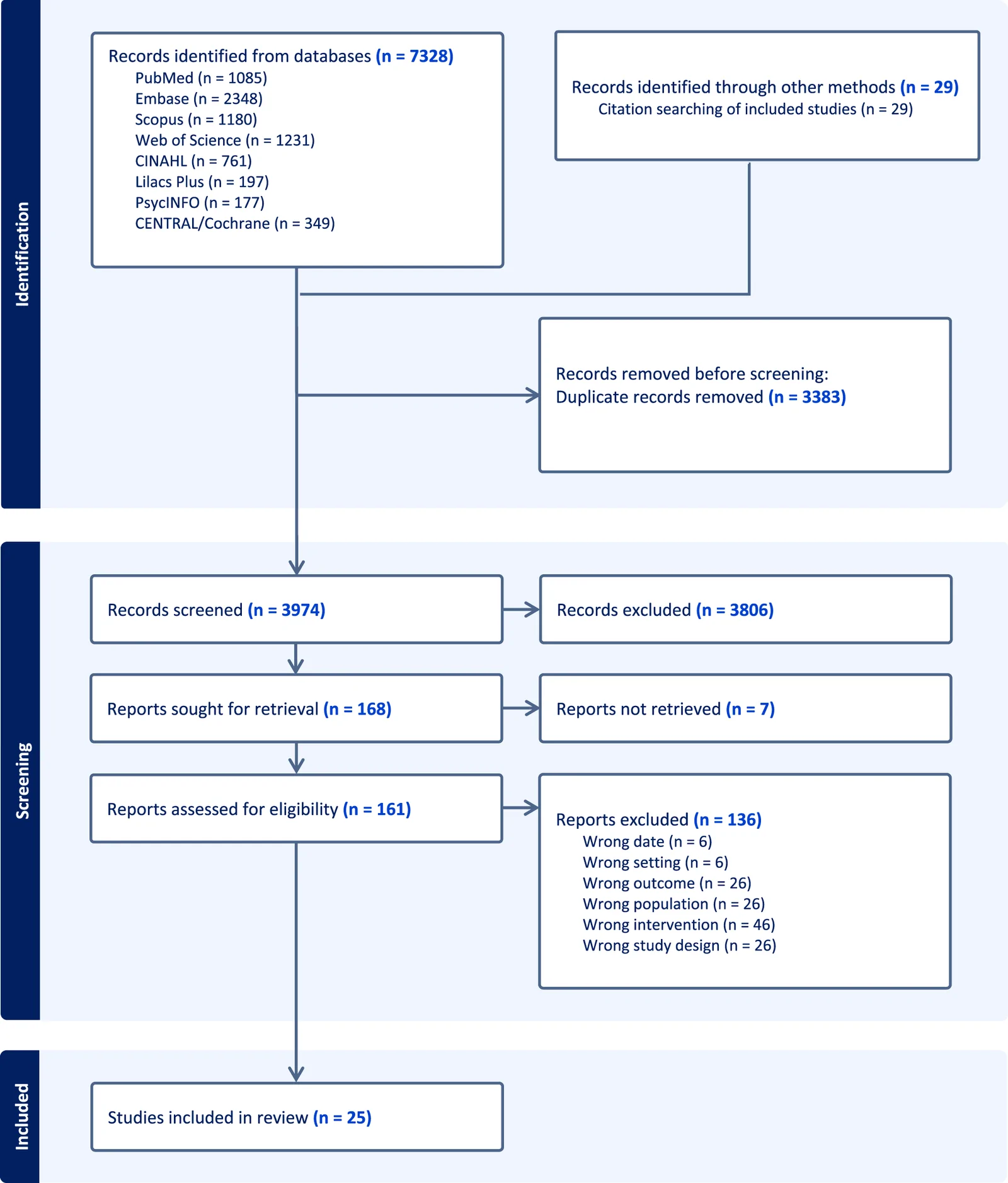
PRISMA 2020 flow diagram. Flow diagram showing the identification, screening, eligibility assessment and inclusion of studies in the systematic review

The database searches identified 7,328 records. In addition, citation searching of the included studies identified 29 additional records, giving a total of 7,357 records. After the removal of 3,383 duplicates, 3,974 records underwent title and abstract screening. Of these, 3,806 were excluded and 168 reports were sought for retrieval. Seven reports could not be obtained, leaving 161 full-text articles for eligibility assessment. A further 136 articles were excluded because of publication date, setting, population, outcome, intervention or study design. Ultimately, 25 studies were included in the systematic review and narrative synthesis (Fig. 1).

### Study characteristics

The 25 included studies comprised 4,653 nurses and used cross-sectional designs [16–40]. The studies were published between 2013 and 2026 and represented 17 countries across four geographical regions. Eleven studies were conducted in Asia, five in South America, five in Africa and four in Europe. No studies from North America or Oceania were identified. The included articles were published in English, Portuguese, Korean or Spanish. Sample sizes ranged from 14 to 1,241 participants. Participants were recruited from a wide range of clinical settings, including emergency departments, intensive and coronary care units, cardiology departments, operating rooms, general medical and surgical wards, and mixed hospital settings.

The studies assessed knowledge of general cardiopulmonary resuscitation (CPR), basic life support (BLS) or advanced life support (ALS). Some studies recruited nurses exclusively, whereas others included mixed professional or nursing-team samples from which nurse-specific data could be extracted.

Most studies were published in the more recent period: 15 (60%) from 2020 onward [16–18, 20, 22–24, 29, 30, 32, 34–36, 38, 39], nine (36%) between 2015 and 2019 [19, 21, 25–28, 33, 37, 40], and one (4%) before 2015 [31].

Five studies used 2010-based recommendations [19, 31, 33, 37, 40], with data collected between 2012 and 2015; nine incorporated the 2015 recommendations or subsequent focused updates [18, 22, 24–27, 32, 36, 38], of which eight reported identifiable data collection periods spanning 2016–2019 [22, 24–27, 32, 36, 38]; and three used the 2020 AHA recommendations [29, 34, 35], all of which collected data in 2023. In the remaining eight studies, the assessment was not clearly linked to a specific AHA/ERC guideline version [16, 17, 20, 21, 23, 28, 30, 39]. None of the included assessments was based on the 2025 AHA/ERC guidelines. The characteristics and principal findings of the included studies are presented in Additional file 2.

### CPR knowledge assessment methods

Considerable heterogeneity was observed in the methods used to assess CPR knowledge. The instruments ranged from seven to 32 knowledge items and included previously developed instruments [17, 28, 39], certification examinations [18], questionnaires adapted from international resuscitation guidelines [19, 22, 24, 29, 31–33, 35–38] and instruments developed specifically for individual studies [16, 20, 21, 23, 25–27, 30, 34, 40].

The domains assessed also varied. Some instruments evaluated general CPR knowledge [17, 19, 20, 23–25, 27, 28, 30, 31, 35, 38–40], whereas others focused on BLS [21, 26, 36], ALS or advanced cardiac life support (ACLS) [16, 18, 34], or a combination of BLS and ALS or ACLS [22, 29, 32, 33, 37]. In studies assessing general CPR knowledge or more than one resuscitation domain, the reported scores generally reflected overall CPR knowledge rather than separate BLS and ALS/ACLS knowledge. Likewise, studies including nurses from different clinical areas generally reported aggregate scores for the overall sample rather than setting-specific data. Although isolated studies reported setting or domain-specific differences, these data were too sparse and heterogeneous to identify a consistent pattern across studies. Therefore, a reliable descriptive comparison between general wards and specialized departments, or between BLS and ALS/ACLS knowledge, was not possible.

Reporting of instrument development and measurement properties was inconsistent. Expert review or content validation was reported in several studies [16, 19, 22, 26, 29, 34, 36, 38], while pilot testing or pretesting was described in others [19, 20, 23, 27, 29, 30, 32–34, 36–38, 40]. Internal consistency was reported in six studies, with Cronbach’s alpha coefficients ranging from 0.62 to 0.90 [16, 23, 26, 28, 32, 34]. Test–retest reliability was reported in a limited number of studies [19, 36, 39, 40]. Other studies provided little or no information regarding the validity or reliability of the instruments used [18, 20, 21, 24, 25, 27, 30, 31, 33, 35, 37].

### Definitions of adequate CPR knowledge

The criteria used to define adequate CPR knowledge differed markedly across studies. Absolute competency thresholds ranged from approximately 50% to more than 85% of correct responses. Five studies used cut-offs close to 50% [16, 23, 33, 37, 38], two used 70% [24, 25], one used 75% [34], four adopted an 80% criterion [18, 32, 35, 39], and two applied thresholds of 85% or higher [22, 27].

Other studies relied on relative or instrument-specific classifications rather than externally established competency standards. These included tercile-based categorization [19] and study-specific score ranges defining low, moderate, average, good or high knowledge [17, 21, 30, 36]. Several studies reported only mean scores, proportions of correct responses or descriptive knowledge levels without specifying a clear pass criterion [20, 26, 28, 29, 31, 40].

Because the instruments, scoring systems, knowledge domains and competency thresholds were not equivalent, the proportions of nurses classified as having adequate knowledge were not considered directly comparable. This heterogeneity precluded quantitative pooling.

### Overall CPR knowledge

CPR knowledge was generally reported as poor, insufficient, moderate or average [17, 20, 21, 26, 28, 30, 32, 34–36]. However, the proportion of nurses meeting the study-defined competency criterion varied substantially according to the threshold applied.

Among the five studies using thresholds of approximately 50%–55.6%, the proportion meeting the criterion ranged from 35.9% to 87.9% [16, 23, 33, 37, 38]. The highest proportion was reported by Damprane et al., who defined good knowledge as at least five correct responses out of nine, equivalent to a threshold of 55.6% [23].

In studies applying thresholds of 70%–75%, the proportion classified as having satisfactory or good knowledge ranged from 26.4% to 42.9% [24, 25, 34]. Among studies applying thresholds of approximately 80% or higher, most reported low pass rates [18, 22, 27, 32, 35, 39]. The proportion meeting the required standard ranged from 0% to 26.8% in five studies [18, 22, 27, 32, 35]. One additional study using an 80% threshold reported that 50% of participants met the criterion [39]. In the two studies applying thresholds of 85% or higher, no participant passed in one study and 4.4% passed in the other [22, 27].

Studies using relative or instrument-specific classifications also reported variable outcomes. Aranzábal-Alegría et al. classified 49.6% of nurses as having good knowledge using the highest tercile of scores rather than an external competency standard [19]. In studies using predefined categories such as low, moderate, average, good or high, participants were most frequently placed in the intermediate categories [17, 21, 30, 36].

Other studies reported mean scores close to or below half of the maximum possible score, including assessments of BLS, ALS and combined BLS/ACLS knowledge [22, 25, 26, 29, 31, 33, 37, 40].

### Previous CPR training

Information on previous CPR training, certification or practical experience was reported in 16 studies [18, 21–25, 28–30, 33–39]. The remaining studies either did not report training data or did not provide results separately for nurses [16, 17, 19, 20, 26, 27, 31, 32, 40].

The proportion of nurses with previous training or certification varied from approximately 14% to 97.1% [21, 28]. Fewer than half of participants had received training or held certification in five studies [21, 29, 34, 36, 38]. Proportions ranging from approximately 53% to 64% were reported in six studies [18, 23–25, 30, 39], while proportions above 90% were reported in three [22, 28, 37]. One study reported that all participants had previous CPR experience and had performed CPR, although formal training was not specified [35].

The type and recency of training were reported inconsistently and included course attendance, BLS or ACLS certification, institutional training requirements and previous practical experience. Knowledge outcomes were not consistently reported according to time since training, precluding a meaningful comparison of knowledge performance by training recency across studies.

### Methodological quality of the included studies

The methodological quality of the 25 included studies was assessed using the nine-item Joanna Briggs Institute Critical Appraisal Checklist for Prevalence Studies (Additional file 3). No study was excluded on the basis of methodological quality. Overall, the studies generally provided adequate descriptions of participants, settings and statistical analyses. However, several recurring limitations were identified.

The most frequent methodological concerns related to sampling procedures, sample-size justification and reporting of response rates. Appropriate sampling procedures were reported in 12 studies, whereas 13 studies did not meet this criterion. Sample-size adequacy was confirmed in 12 studies and was unclear in 13. Reporting of response rates was also incomplete, with 13 studies reporting an adequate response rate or appropriate management of non-response and 12 rated as unclear.

Regarding measurement quality, 19 studies were rated as using a valid method to assess CPR knowledge, whereas six were rated as unclear. This reflected incomplete reporting of the development, validation or reliability of several knowledge instruments. The number of JBI criteria rated as “yes” ranged from five to nine across studies, indicating variability in methodological quality. These appraisal findings were considered when interpreting the confidence in and comparability of the findings.

## Discussion

### Principal findings

This review identified two closely related findings. Hospital nurses’ CPR knowledge was frequently incomplete or intermediate, while the reported level depended substantially on the assessment instrument, resuscitation domain and competency threshold used. This methodological variability prevented direct comparison and precluded estimation of an overall prevalence of adequate knowledge.

Despite these limitations, the findings remain clinically relevant because nurses’ knowledge contributes to institutional preparedness for in-hospital cardiac arrest. Nurses are expected to recognize clinical deterioration, identify cardiac arrest and initiate time-critical interventions [8, 9]. Knowledge should therefore be considered a component of professional nursing competence and patient safety rather than solely an individual educational outcome.

### CPR knowledge as a core component of professional preparedness

High-quality CPR depends on multiple factors, including psychomotor skills, communication, teamwork, confidence, familiarity with equipment and organizational support [12, 41]. However, adequate knowledge provides the cognitive basis for recognizing cardiac arrest and applying the appropriate sequence of interventions [42].

The frequent finding of incomplete or intermediate knowledge suggests that some nurses may lack part of the cognitive foundation required for a timely, guideline-based response. Although questionnaire scores cannot be interpreted as a direct measure of clinical performance, knowledge deficits may restrict the effective application of technical and non-technical skills.

Previous reviews have shown that CPR knowledge and skills deteriorate after training and that retention is influenced by retraining frequency, opportunities for practice and educational methods [43–45]. An integrative review of ward nurses’ BLS experiences also identified barriers related to staff interaction, confidence, fear of causing harm or litigation, defibrillation and training [41]. CPR preparedness should therefore be understood as a multidimensional competence in which knowledge underpins decision-making, technical performance and teamwork.

### Assessment instruments and competency thresholds

One of the principal contributions of this review is the substantial variability in CPR knowledge assessment. Instruments differed in content, guideline source, resuscitation domain, scoring system and competency threshold. Some focused on cardiac arrest recognition and initial CPR, whereas others included advanced content. Differences between studies may therefore reflect the scope and difficulty of the assessment rather than genuine differences in nurses’ knowledge. Temporal differences across successive AHA/ERC guideline cycles should also be considered, as guideline updates may influence educational content and assessment instruments.

Evidence supporting measurement quality was also inconsistent, with limited reporting of validity, reliability and temporal stability. Competency thresholds were frequently study specific and rarely justified using professional or empirical criteria, making classifications such as poor, moderate or good difficult to compare. The importance of demonstrating and transparently reporting the psychometric quality of instruments has also been emphasized in nursing research [46].

This lack of standardization limits the interpretation and comparison of reported knowledge levels. It also prevented reliable exploration of whether knowledge differed according to clinical area or resuscitation domain. Most studies either recruited nurses from several hospital departments and reported aggregate scores or focused on single settings, while BLS and ALS/ACLS content was often combined within overall CPR knowledge scores. Future work should therefore develop and validate standardized, context-adapted instruments aligned with current resuscitation guidance and clearly defined nursing responsibilities. Such instruments should distinguish core BLS knowledge from advanced, role-specific content and report outcomes separately by clinical setting and resuscitation domain, using formally justified competency thresholds. This would improve comparability and provide a consistent basis for identifying specific educational needs among hospital nurses [42, 46].

### Implications for nursing education and research

Several included studies reported higher knowledge among nurses with previous or recent CPR training [18, 29, 33, 34, 36–39]. This is consistent with evidence that training and retraining improve knowledge and practical performance, although learning may decline without periodic reinforcement [47]. However, high levels of previous training did not consistently correspond to adequate knowledge [22, 28, 37], indicating that course completion alone may not be sufficient to demonstrate current knowledge or overall preparedness for in-hospital cardiac arrest. Differences in course content, time since training, opportunities for practice, subsequent assessment and competency thresholds may partly explain this inconsistency.

These findings are consistent with the direction of successive resuscitation education guidelines. The 2015 AHA and ERC guidelines explicitly addressed resuscitation education and implementation [48, 49], while the 2020 AHA education-science guidelines emphasized strategies such as deliberate practice, booster training, spaced learning and feedback to support knowledge and skill acquisition and retention [50]. The 2021 and 2025 ERC education guidelines have continued to emphasize repeated and spaced learning, feedback-informed training and educational approaches adapted to healthcare professionals and their clinical context [12, 42]. Together, these recommendations support moving beyond isolated courses or infrequent certification cycles towards educational strategies designed to maintain competence over time. Potential approaches include low-dose, high-frequency training, e-learning, simulation and structured debriefing, although the optimal combination remains uncertain [12, 42, 51]. Training should preserve a common core of resuscitation knowledge while also being adapted to nurses’ scope of practice and clinical context.

Knowledge assessment should be used to identify specific learning needs and guide targeted education rather than simply classify nurses as passing or failing. Periodic assessment could detect deficits in cardiac arrest recognition, chest-compression quality, ventilation, defibrillation or advanced algorithms, allowing educational interventions to address the domains most relevant to nurses’ clinical practice.

Future research should prioritize the development and validation of a standardized questionnaire with a common BLS section and a separate advanced section for role-specific knowledge. Competency thresholds should be formally justified, and longitudinal studies are needed to determine how knowledge changes over time, establish appropriate reassessment and recertification intervals, and identify which educational methods produce the most sustained improvements.

### Implications for patient safety and institutional preparedness

The frequently incomplete or intermediate knowledge levels identified in this review may have implications for patient safety, particularly because nurses are often involved in the early recognition and initial response to in-hospital cardiac arrest. Although knowledge alone does not ensure high-quality resuscitation, deficits may contribute to incorrect priorities, delayed interventions or poor adherence to recommended algorithms. The potential clinical relevance of CPR knowledge is supported by evidence showing an association between higher advanced life support knowledge among healthcare professionals and greater return of spontaneous circulation after in-hospital cardiac arrest [52].

Nursing knowledge is particularly important within the multidisciplinary response. Nurses sustain essential bedside interventions throughout resuscitation and may act as first responders, team members and clinical or organizational leaders [8, 53]. In top-performing hospitals, nurses are actively integrated into dedicated resuscitation teams characterized by clearly defined roles, effective communication, strong leadership and regular mock-code training [54].

Variation in survival between hospitals suggests that outcomes depend partly on how consistently resuscitation care is organized and delivered. Practices associated with better survival include adequate staff preparation, monitoring interruptions in chest compressions and systematic review of cardiac-arrest events [55]. The knowledge deficits identified in this review may therefore represent not only an educational issue, but also a potential weakness within the wider resuscitation system.

Maintaining an appropriately knowledgeable nursing workforce should be understood as a shared responsibility. Nurses should engage actively in continuing professional development, while institutions should provide regular and accessible training together with periodic assessment to identify knowledge gaps and implement targeted educational interventions to address them. This shared responsibility, supported by reliable team processes and effective organization of the resuscitation response, may reduce avoidable variation in care and contribute to improved survival and neurological outcomes [54, 55].

### Methodological quality of the included studies

The methodological quality of the included studies should be considered when interpreting the findings. Although most studies adequately described their participants, settings and analyses, recurrent limitations were identified in sampling procedures, sample-size justification, response-rate reporting and measurement quality.

Many studies used non-probability sampling or did not clearly justify their sample size, which may limit representativeness and precision. Incomplete reporting of response rates also makes it difficult to assess the potential influence of non-response bias. In addition, several studies provided limited information on the development, validity or reliability of the knowledge instruments used, reducing confidence in the comparability and accuracy of reported knowledge levels.

These limitations do not invalidate the overall finding that CPR knowledge was frequently incomplete or intermediate. However, they require cautious interpretation of the magnitude of the reported deficits and reinforce the need for more robust primary studies using transparent sampling strategies, justified sample sizes, adequate response-rate reporting and valid, reliable assessment instruments. Improving methodological quality would strengthen the evidence base and allow more reliable identification of educational priorities for hospital nurses.

### Strengths and limitations

This review synthesized evidence specifically on objectively assessed CPR knowledge among hospital nurses. It included studies across different hospital settings and languages, and examined not only knowledge outcomes but also assessment instruments, competency thresholds and methodological quality. This approach allowed the findings to be interpreted in relation to how knowledge was measured, rather than treating all reported scores as directly comparable.

Several limitations should also be acknowledged. Substantial differences in instruments, resuscitation domains, scoring systems and competency thresholds precluded quantitative pooling and limited direct comparison between studies. These differences also limited comparisons across countries and regions, particularly because the included studies were unevenly distributed geographically, with no eligible studies identified from North America or Oceania.

Grey literature sources, including theses, conference proceedings, and institutional reports, were not searched, and study authors were not contacted to clarify missing or unclear information. Consequently, reporting and publication bias cannot be ruled out, and potentially relevant unpublished evidence, nurse-specific data, or additional details on knowledge outcomes, assessment instruments, and previous training may have been missed. Baseline data from interventional and pre–post studies were excluded to maintain a consistent observational evidence base and avoid including samples selected specifically for educational interventions; however, this decision may have omitted some relevant information on nurses’ CPR knowledge before training.

Finally, this review focused on theoretical knowledge among nurses caring for adults in hospital settings. Practical skills, confidence, attitudes, teamwork, clinical performance and patient outcomes were outside its scope and may be addressed in future research. The findings therefore represent one component of professional resuscitation competence and should not be interpreted as a direct measure of the quality of CPR delivered in clinical practice.

## Conclusions

Hospital nurses’ CPR knowledge was frequently incomplete or intermediate, although the reported levels varied substantially according to the assessment instrument, resuscitation domain and competency threshold used. This lack of standardization limits comparison across studies and prevents estimation of an overall prevalence of adequate knowledge.

CPR knowledge should be regarded as a core component of professional nursing competence, institutional preparedness and patient safety. Hospitals should consider regular and accessible CPR training, periodic assessment to identify knowledge gaps and targeted educational interventions to address them. Future research should prioritize validated instruments, formally justified competency thresholds and longitudinal studies to determine how knowledge changes over time and which educational strategies produce sustained improvements.

## Supplementary Information

Below is the link to the electronic supplementary material.

Supplementary Material 1: Additional file 1 (.docx). Search strategies. Complete search strategies used in all bibliographic databases.

Supplementary Material 2: Additional file 2 (.xlsx). Characteristics of the included studies. Study characteristics, assessment methods, knowledge findings, competency thresholds, and previous CPR training.

Supplementary Material 3: Additional file 3 (.xlsx). Methodological quality assessment. Results of the JBI critical appraisal of the 25 included studies.

Supplementary Material 4: Additional file 4 (.docx). PRISMA 2020 checklist. Completed PRISMA 2020 checklist indicating where each item is reported in the manuscript.

## Data Availability

All data supporting the findings of this study are available within the paper and its Supplementary Information.

## Declarations

### AI and AI-assisted technologies in the writing process

During the preparation of this work, the authors used ChatGPT (OpenAI) to support language editing and translation. After using this tool, the authors reviewed and edited the content as needed and take full responsibility for the content of the manuscript. No AI tool was used to generate, analyze or interpret data.

## Abbreviations

A-B-C: Airway-breathing-chest compressions
ACLS: Advanced cardiac life support
AHA: American Heart Association
ALS: Advanced life support
BLS: Basic life support
C-A-B: Chest compressions-airway-breathing
CINAHL: Cumulative Index to Nursing and Allied Health Literature
CPR: Cardiopulmonary resuscitation
ERC: European Resuscitation Council
JBI: Joanna Briggs Institute
LILACS: Latin American and Caribbean Health Sciences Literature
MeSH: Medical Subject Headings
PRISMA: Preferred Reporting Items for Systematic Reviews and Meta-Analyses
PROSPERO: International Prospective Register of Systematic Reviews
