# Supplementary material for: Hospital nurses’ knowledge of adult cardiopulmonary resuscitation: a systematic review
Source: BMC Nurs. 2026 Oct 17;25:1097. doi: 10.1186/s12912-026-05483-1 (PMC13757171; doi:10.1186/s12912-026-05483-1)
Supplement: Supplementary file 1 [file 12912_2026_5483_MOESM1_ESM.docx]

**Additional file 1. Search strategies**

**Description**: Complete search strategies used in the bibliographic databases included in the systematic review.

**Limits**: All database searches were conducted on March 15, 2026. No language restrictions were applied. Eligible studies were limited to those published between January 1, 2010 and March 15, 2026, according to the eligibility criteria reported in the manuscript.

**PubMed**

((Cardiopulmonary Resuscitation[mh] OR Advanced Cardiac Life Support[mh] OR Basic Cardiac Life Support[tiab] OR Cardiopulmonary Resuscitation[tiab] OR Cardio Pulmonary Resuscitation[tiab] OR Cardio-Pulmonary Resuscitation[tiab] OR Code Blue[tiab] OR CPR[tiab] OR BLS[tiab] OR ACLS[tiab] OR Life Support, Basic Cardiac[tiab] OR Resuscitation, Cardio-Pulmonary[tiab] OR Resuscitation, Cardiopulmonary[tiab] OR Resuscitation[tiab] OR Life Support[tiab] OR Advanced Cardiac Life Support[tiab] OR Cardiac Life Support, Advanced[tiab] OR Life Support, Advanced Cardiac[tiab]) OR (Heart Arrest[mh] OR Heart Arrest[tiab] OR Arrest, Cardiac[tiab] OR Arrest, Cardiopulmonary[tiab] OR Arrest, Heart[tiab] OR Asystol*[tiab] OR Cardiac Arrest[tiab] OR Cardiopulmonary Arrest[tiab] OR in-hospital cardiac arrest[tiab] OR IHCA[tiab])) AND (Knowledge[mh] OR Knowledge[tiab] OR awareness[tiab] OR understanding[tiab]) AND (Nurses[mh] OR Nursing staff[mh] OR Nursing[mh] OR Nursing, Team[mh] OR Nursing staff, hospital[mh] OR Nurse’s role[mh] OR Nurs*[tiab] OR Nurs*, Registered[tiab] OR Nursing Personnel[tiab] OR Personnel, Nursing[tiab] OR Registered Nurs*[tiab] OR Nursing[tiab] OR Nursing Staff*[tiab] OR Staff*, Nursing[tiab] OR Nursing, Team[tiab] OR Nursing Team[tiab] OR Hospital Nursing Staff*[tiab] OR Nursing Staff*, Hospital[tiab] OR Staff*, Hospital Nursing[tiab] OR hospital nurse[tiab] OR Nursing staff*[tiab] OR Nurs* Role*[tiab] OR Nurs* Scope, Practice[tiab] OR Practice Nurse's Scope*[tiab] OR Role*, Nurs*[tiab])

**Scopus**

ABS ( ( ( "Cardiopulmonary Resuscitation" OR "Advanced Cardiac Life Support" OR "Basic Cardiac Life Support" OR "Cardio Pulmonary Resuscitation" OR "Cardio-Pulmonary Resuscitation" OR "Code Blue" OR CPR OR BLS OR ACLS OR "Life Support, Basic Cardiac" OR "Resuscitation, Cardio-Pulmonary" OR "Resuscitation, Cardiopulmonary" OR Resuscitation OR "Life Support" OR "Advanced Cardiac Life Support" OR "Cardiac Life Support, Advanced" OR "Life Support, Advanced Cardiac" ) OR ( "Heart Arrest" OR "Arrest, Cardiac" OR "Arrest, Cardiopulmonary" OR "Arrest, Heart" OR Asystol* OR "Cardiac Arrest" OR "Cardiopulmonary Arrest" OR "in-hospital cardiac arrest" OR IHCA ) ) AND ( Knowledge OR awareness OR understanding ) AND ( Nursing OR "Nursing staff, hospital" OR "Nurse’s role" OR Nurs* OR "Nurs*, Registered" OR "Nursing Personnel" OR "Personnel, Nursing" OR "Registered Nurs*" OR "Staff*, Nursing" OR "Nursing, Team" OR "Nursing Team" OR "Hospital Nursing Staff*" OR "Staff*, Hospital Nursing" OR "hospital nurse" OR "Nursing staff" OR "Nurs* Role*" OR "Nurs* Scope, Practice" OR "Practice Nurse's Scope*" OR "Role*, Nurs*" ) )

**CINAHL**

XB (("Cardiopulmonary Resuscitation" OR "Advanced Cardiac Life Support" OR "Basic Cardiac Life Support" OR "Cardio Pulmonary Resuscitation" OR "Cardio-Pulmonary Resuscitation" OR "Code Blue" OR CPR OR BLS OR ACLS OR "Life Support, Basic Cardiac" OR "Resuscitation, Cardio-Pulmonary" OR "Resuscitation, Cardiopulmonary" OR Resuscitation OR "Life Support" OR "Advanced Cardiac Life Support" OR "Cardiac Life Support, Advanced" OR "Life Support, Advanced Cardiac") OR ("Heart Arrest" OR "Arrest, Cardiac" OR "Arrest, Cardiopulmonary" OR "Arrest, Heart" OR Asystol* OR "Cardiac Arrest" OR "Cardiopulmonary Arrest" OR "in-hospital cardiac arrest" OR IHCA)) AND (Knowledge OR awareness OR understanding) AND (Nursing OR "Nursing staff, hospital" OR "Nurse’s role" OR Nurs* OR "Nurs*, Registered" OR "Nursing Personnel" OR "Personnel, Nursing" OR "Registered Nurs*" OR "Staff*, Nursing" OR "Nursing, Team" OR "Nursing Team" OR "Hospital Nursing Staff*" OR "Staff*, Hospital Nursing" OR "hospital nurse" OR "Nursing staff" OR "Nurs* Role*" OR "Nurs* Scope, Practice" OR "Practice Nurse's Scope*" OR "Role*, Nurs*")

Note: XB refers to the title and abstract search field as returned by CINAHL/EBSCOhost.

**Cochrane Central Register of Controlled Trials (CENTRAL)**

(("Cardiopulmonary Resuscitation" OR "Advanced Cardiac Life Support" OR "Basic Cardiac Life Support" OR "Cardiopulmonary Resuscitation" OR "Cardio Pulmonary Resuscitation" OR "Cardio-Pulmonary Resuscitation" OR "Code Blue" OR CPR OR BLS OR ACLS OR "Life Support, Basic Cardiac" OR "Resuscitation, Cardio-Pulmonary" OR "Resuscitation, Cardiopulmonary" OR Resuscitation OR "Life Support" OR "Advanced Cardiac Life Support" OR "Cardiac Life Support, Advanced" OR "Life Support, Advanced Cardiac") OR ("Heart Arrest" OR "Arrest, Cardiac" OR "Arrest, Cardiopulmonary" OR "Arrest, Heart" OR Asystol* OR "Cardiac Arrest" OR "Cardiopulmonary Arrest" OR "in-hospital cardiac arrest" OR IHCA)) AND (Knowledge OR awareness OR understanding) AND (Nursing OR "Nursing staff, hospital" OR "Nurse’s role" OR Nurs* OR "Nurs*, Registered" OR "Nursing Personnel" OR "Personnel, Nursing" OR "Registered Nurs*" OR "Staff*, Nursing" OR "Nursing, Team" OR "Nursing Team" OR "Hospital Nursing Staff*" OR "Staff*, Hospital Nursing" OR "hospital nurse" OR "Nursing staff" OR "Nurs* Role*" OR "Nurs* Scope, Practice" OR "Practice Nurse's Scope*" OR "Role*, Nurs*") in Title, Abstract, or Keyword

**APA PsycINFO (Ovid)**

(("Cardiopulmonary Resuscitation" or "Advanced Cardiac Life Support" or "Basic Cardiac Life Support" or "Cardio Pulmonary Resuscitation" or "Cardio-Pulmonary Resuscitation" or "Code Blue" or CPR or BLS or ACLS or "Life Support, Basic Cardiac" or "Resuscitation, Cardio-Pulmonary" or "Resuscitation, Cardiopulmonary" or Resuscitation or "Life Support" or "Advanced Cardiac Life Support" or "Cardiac Life Support, Advanced" or "Life Support, Advanced Cardiac" or ("Heart Arrest" or "Arrest, Cardiac" or "Arrest, Cardiopulmonary" or "Arrest, Heart" or Asystol* or "Cardiac Arrest" or "Cardiopulmonary Arrest" or "in-hospital cardiac arrest" or IHCA)) and (Knowledge or awareness or understanding) and (Nursing OR "Nursing staff, hospital" OR "Nurse’s role" OR Nurs* OR "Nurs*, Registered" OR "Nursing Personnel" OR "Personnel, Nursing" OR "Registered Nurs*" OR "Staff*, Nursing" OR "Nursing, Team" OR "Nursing Team" OR "Hospital Nursing Staff*" OR "Staff*, Hospital Nursing" OR "hospital nurse" OR "Nursing staff" OR "Nurs* Role*" OR "Nurs* Scope, Practice" OR "Practice Nurse's Scope*" OR "Role*, Nurs*")).mp. [mp=title, abstract, heading word, table of contents, key concepts, original title, tests & measures, mesh word]

**LILACS Plus**

(("Cardiopulmonary Resuscitation" OR "Advanced Cardiac Life Support" OR "Basic Cardiac Life Support" OR "Cardio Pulmonary Resuscitation" OR "Cardio-Pulmonary Resuscitation" OR "Code Blue" OR CPR OR BLS OR ACLS OR "Life Support, Basic Cardiac" OR "Resuscitation, Cardio-Pulmonary" OR "Resuscitation, Cardiopulmonary" OR Resuscitation OR "Life Support" OR "Advanced Cardiac Life Support" OR "Cardiac Life Support, Advanced" OR "Life Support, Advanced Cardiac") OR ("Heart Arrest" OR "Arrest, Cardiac" OR "Arrest, Cardiopulmonary" OR "Arrest, Heart" OR Asystol* OR "Cardiac Arrest" OR "Cardiopulmonary Arrest" OR "in-hospital cardiac arrest" OR IHCA)) AND (Knowledge OR awareness OR understanding) AND (Nursing OR "Nursing staff, hospital" OR "Nurse’s role" OR Nurs* OR "Nurs*, Registered" OR "Nursing Personnel" OR "Personnel, Nursing" OR "Registered Nurs*" OR "Staff*, Nursing" OR "Nursing, Team" OR "Nursing Team" OR "Hospital Nursing Staff*" OR "Staff*, Hospital Nursing" OR "hospital nurse" OR "Nursing staff" OR "Nurs* Role*" OR "Nurs* Scope, Practice" OR "Practice Nurse's Scope*" OR "Role*, Nurs*")

**Web of Science**

(("Cardiopulmonary Resuscitation" OR "Advanced Cardiac Life Support" OR "Basic Cardiac Life Support" OR "Cardio Pulmonary Resuscitation" OR "Cardio-Pulmonary Resuscitation" OR "Code Blue" OR CPR OR BLS OR ACLS OR "Life Support, Basic Cardiac" OR "Resuscitation, Cardio-Pulmonary" OR "Resuscitation, Cardiopulmonary" OR Resuscitation OR "Life Support" OR "Advanced Cardiac Life Support" OR "Cardiac Life Support, Advanced" OR "Life Support, Advanced Cardiac") OR ("Heart Arrest" OR "Arrest, Cardiac" OR "Arrest, Cardiopulmonary" OR "Arrest, Heart" OR Asystol* OR "Cardiac Arrest" OR "Cardiopulmonary Arrest" OR "in-hospital cardiac arrest" OR IHCA)) AND (Knowledge OR awareness OR understanding) AND (Nursing OR "Nursing staff, hospital" OR "Nurse’s role" OR Nurs* OR "Nurs*, Registered" OR "Nursing Personnel" OR "Personnel, Nursing" OR "Registered Nurs*" OR "Staff*, Nursing" OR "Nursing, Team" OR "Nursing Team" OR "Hospital Nursing Staff*" OR "Staff*, Hospital Nursing" OR "hospital nurse" OR "Nursing staff" OR "Nurs* Role*" OR "Nurs* Scope, Practice" OR "Practice Nurse's Scope*" OR "Role*, Nurs*")

**Embase (Ovid)**

(("Cardiopulmonary Resuscitation" or "Advanced Cardiac Life Support" or "Basic Cardiac Life Support" or "Cardio Pulmonary Resuscitation" or "Cardio-Pulmonary Resuscitation" or "Code Blue" or CPR or BLS or ACLS or "Life Support, Basic Cardiac" or "Resuscitation, Cardio-Pulmonary" or "Resuscitation, Cardiopulmonary" or Resuscitation or "Life Support" or "Advanced Cardiac Life Support" or "Cardiac Life Support, Advanced" or "Life Support, Advanced Cardiac" or ("Heart Arrest" or "Arrest, Cardiac" or "Arrest, Cardiopulmonary" or "Arrest, Heart" or Asystol* or "Cardiac Arrest" or "Cardiopulmonary Arrest" or "in-hospital cardiac arrest" or IHCA)) and (Knowledge or awareness or understanding)).mp. and (Nursing OR "Nursing staff, hospital" OR "Nurse’s role" OR Nurs* OR "Nurs*, Registered" OR "Nursing Personnel" OR "Personnel, Nursing" OR "Registered Nurs*" OR "Staff*, Nursing" OR "Nursing, Team" OR "Nursing Team" OR "Hospital Nursing Staff*" OR "Staff*, Hospital Nursing" OR "hospital nurse" OR "Nursing staff" OR "Nurs* Role*" OR "Nurs* Scope, Practice" OR "Practice Nurse's Scope*" OR "Role*, Nurs*").ab,hw,kf,kw,ti.
